# Artificial Intelligence in Oral and Maxillofacial Surgery: Integrating Clinical Innovation and Workflow Optimization

**DOI:** 10.3390/jcm15020427

**Published:** 2026-01-06

**Authors:** Majeed Rana, Andreas Sakkas, Matthias Zimmermann, Maurício Kostyuk, Guilherme Schwarz

**Affiliations:** 1 Department of Oral and Maxillofacial Surgery, University Hospital Ulm, Albert-Einstein University Ulm, 89081 Ulm, Germany; 2 Department of Oral and Maxillofacial Surgery, University Hospital Vienna, Medical University Vienna, 1090 Vienna, Austria; 3 The Wharton School, University of Pennsylvania, 3733 Spruce Street, Philadelphia, PA 19104, USA; mauricio@eaglemidia.com; 4 Stanford University School of Medicine, Stanford University, 291 Campus Drive, Li Ka Shing Building, Stanford, CA 94305-5101, USA; guilherme@eaglemidia.com

**Keywords:** artificial intelligence, oral and maxillofacial surgery, virtual surgical planning, clinical decision support, workflow optimization, patient engagement

## Abstract

**Objective**: The objective of this study is to synthesize and critically appraise how artificial intelligence (AI) is being integrated into oral and maxillofacial surgery (OMFS). This review’s novel contribution is to jointly map clinical applications (diagnostics, virtual surgical planning, intraoperative guidance) and operational uses (triage, scheduling, documentation, patient communication), quantifying evidence and validation status to provide practice-oriented guidance for adoption. **Study Design**: A narrative review of the recent literature and expert analysis, supplemented by illustrative multicenter implementation data from OMFS practice, was carried out. **Results**: AI demonstrates high performance in radiographic analysis and virtual planning (up to 96% predictive accuracy and sub-millimeter soft-tissue simulation error), with clinical reports of shorter planning times and more efficient patient communication. Early deployments in OMFS clinics have increased appointment bookings, while maintaining high patient satisfaction, and reduced the administrative burden. Remaining challenges include data quality, explainability, and limited multicenter and pediatric validation, which constrain generalizability and require clinician oversight. **Conclusions**: AI offers substantive benefits across the OMFS care continuum—improving diagnostic accuracy, surgical planning, and patient engagement while streamlining workflows. Responsible adoption depends on transparent validation, data governance, and targeted training, with attention to cost-effectiveness. Immediate priorities include standardized reporting of quantitative outcomes (e.g., sensitivity, specificity, time saved) and prospective multicenter studies, ensuring that AI augments—rather than replaces—human-centered care.

## 1. Introduction

Artificial intelligence (AI) is rapidly transforming healthcare, including oral and maxillofacial surgery (OMFS), and should be understood as part of the broader digitalization of surgery that also encompasses robotics, virtual surgical planning (VSP), and telemedicine [1]. Within this trend, AI brings unique capabilities in radiographic analysis, predictive modeling, workflow optimization, and patient communication. While early studies demonstrate high accuracy of AI in diagnostics and planning—such as up to 96% predictive accuracy in surgical need determination and sub-millimeter precision in facial simulations [2]—a clear gap exists in the literature regarding its practical integration into routine OMFS workflows, particularly in patient engagement and administrative efficiency. Most current reports focus on technical capabilities or isolated tasks, lacking comprehensive synthesis of how AI can address persistent clinical challenges such as patient communication barriers, high administrative burdens, and inefficiencies that limit patient-facing care [3,4,5,6].

A narrative review is necessary now to bridge this gap by contextualizing emerging AI technologies within the specific demands of OMFS practice [3,7,8]. Given the increasing complexity of surgical care, the growing patient volume, and the pressure to improve outcomes while maintaining human-centered care, evaluating AI’s role beyond image analysis—into scheduling, patient triage, and communication—is essential. This review synthesizes current evidence and technological capabilities, critically examining their implications for OMFS while highlighting limitations, ethical considerations, and the need for thoughtful, clinician-guided implementation to enhance care delivery without compromising the patient–surgeon relationship. Compared with previous systematic or scoping reviews on AI in oral and maxillofacial surgery, which have primarily focused on technical validation or single domains such as image segmentation or implant design, the present narrative review provides an integrative synthesis across the full spectrum of clin ical and operational applications. It uniquely combines diagnostic, surgical, and administrative perspectives and includes ethical and implementation considerations that are often absent from earlier reviews. By addressing this interdisciplinary gap, the review aims to support both clinical decision-making and institutional adoption strategies for responsible AI integration in OMFS.

## 2. Materials and Methods

We conducted a narrative literature review and expert consultation to gather information on AI applications relevant to OMFS. A broad search of peer-reviewed publications published up to 2025 was performed using databases such as PubMed and Scopus, as well as professional reports and guidelines on health AI. The search covered literature from 2010 to 2025 to ensure inclusion of both foundational and recent developments. Key search terms included combinations of “artificial intelligence,” “oral and maxillofacial surgery,” “dentistry,” “surgical planning,” “patient engagement,” “workflow,” “natural language processing,” and “machine learning.” Two authors (M.R. and A.S.) independently conducted the literature search to ensure comprehensive coverage and minimize selection bias. Discrepancies in study inclusion were discussed and resolved through consensus with a third reviewer (M.Z.), who verified the final selection. The search strategy employed Boolean operators (“A_I” OR “artificial intelligence”) AND (“oral and maxillofacial surgery”) AND (“planning” OR “workflow” OR “communication”), applied across PubMed and Scopus databases. This calibration process ensured consistency and 85 reproducibility in study identification.

Studies were included if they addressed AI applications in OMFS or dentistry, or if they contributed relevant insights from general medicine, surgical disciplines, or healthcare management. Exclusion criteria were non-peer-reviewed opinion pieces, outdated publications (prior to 2010), and articles lacking relevance to clinical or operational contexts in healthcare.

We prioritized studies in OMFS and dentistry but also incorporated findings from general medicine and healthcare management when applicable. In addition, industry whitepapers and conference proceedings were reviewed to capture the latest developments (for example, American Dental Association reports on AI, and technology surveys in healthcare operations). We supplemented literature findings with illustrative real-world examples, including multicenter observational data from an AI implementation in OMFS clinics, to provide concrete evidence of impact. A total of 64 publications met the inclusion criteria. These comprised 32 clinical validation studies, 18 pilot or feasibility studies, and 14 conceptual or framework papers. Two authors (M.R. and A.S.) independently conducted the database searches and screened the retrieved articles. Any disagreement regarding inclusion was resolved through consensus discussion with a third reviewer (M.Z.). This calibration process ensured consistency in study selection and minimized potential bias.

A narrative review approach was deliberately chosen instead of a systematic review. While systematic reviews are suited for narrowly defined clinical questions with homogeneous datasets, they are less effective when the evidence base is heterogeneous and rapidly evolving, as is the case with AI in OMFS [3,7,8]. A narrative review allows for broader integration of diverse study designs, expert perspectives, and emerging insights, enabling a more comprehensive synthesis of both clinical and operational applications. This approach provides added value by combining critical appraisal of published evidence with contextual interpretation and practical commentary on the integration of AI into OMFS practice.

Each co-author contributed their domain expertise—clinical surgery (M.R., A.S., M.Z.) and healthcare AI implementation (M.K., G.S.)—to interpret findings and ensure a balanced discussion. Throughout the article, we have cited data and specific studies to support our statements, and we have explicitly noted the current limitations of the evidence base.

## 3. Results

### 3.1. Imaging and Planning

AI in Surgical Imaging, Planning, and Reconstruction: One major domain of AI application in OMFS is in preoperative planning and surgical simulation. OMFS relies heavily on imaging (such as panoramic radiographs, cone-beam CT, CT/MRI, and 3D facial scans) for diagnosis and treatment planning. AI-powered image analysis can augment the surgeon’s capabilities by enhancing image quality, identifying anatomical landmarks, and segmenting structures of interest. For instance, AI algorithms have been developed to automatically segment maxillofacial CT scans, mapping bony defects or tumors with high precision [1,9]. In tumor resection planning, such AI-assisted segmentation has demonstrated notable reductions in manual workload for clinicians and improved consistency in identifying margins, especially in challenging cases like mandibular tumors [9]. By training on large datasets of annotated images, deep learning models can learn to delineate anatomical structures (mandibular canal, sinus boundaries, tumor extents) faster and potentially as accurately as human experts. Recent AI-based imaging research has further demonstrated high precision in detecting anatomic variations of the mandible and maxilla that are clinically relevant to surgical safety and preoperative planning (DOI: 10.1038/s41598-025-93250-8). This not only saves time but can also serve as a reliable second check, reducing the risk of oversight.

Building on segmentation and modeling, AI has been integrated into virtual surgical planning (VSP) workflows. In orthognathic (jaw corrective) surgery and facial trauma reconstruction, VSP allows surgeons to “practice” the surgery on a computer model and design cutting guides or implants before entering the operating room. AI is rapidly evolving in this space by improving the speed and accuracy of planning. One example is the use of generative models to assist in designing patient-specific implants: by analyzing a patient’s CT data and comparing it to thousands of prior cases, an AI system can propose an initial implant design or bone graft contour that optimally fits the defect [10]. In our experience, integrating AI into the planning of mandibular reconstructions with selective laser melted (3D-printed) titanium implants has enhanced the fit and functionality of these patient-specific prosthetics, reducing the need for intraoperative adjustments. Rana et al. [11] reported that over one hundred patient-specific orbital floor reconstructions have been successfully completed using AI-enhanced image datasets from CT/MRI to guide implant design [10,12]—an example of how routine clinical workflows are already benefitting from AI-driven personalization. Deep learning algorithms can also assist in predicting surgical outcomes from virtual plans. For instance, AI models have been used to simulate facial soft tissue changes resulting from jaw repositioning surgery, with one study citing sub-millimeter accuracy (~1 mm error) in these predictions [2]. Such simulations help both surgeons and patients visualize post-operative appearance, improving informed consent and satisfaction with results.

Beyond planning, AI technologies are making inroads into intraoperative assistance. Augmented reality (AR) and computer vision, often empowered by AI, can guide surgeons during procedures. A recent study demonstrated an AR system for third molar (wisdom tooth) extractions that overlays the 3D location of the impacted tooth and vital structures on the surgeon’s view in real time [3]. This system relied on AI-driven image processing of the CBCT scan to align the virtual overlay with the patient, and it significantly improved the surgeon’s confidence and precision in a difficult impaction case [3]. Likewise, AI-enhanced navigation systems have been piloted for orbital fracture reconstructions, where an algorithm can continuously track surgical instruments relative to the planned implant position, helping correct any deviations on the fly [1]. These intraoperative tools remain in early stages, but they illustrate the potential for real-time AI guidance to enhance surgical accuracy and safety (Table 1).

AI is also being explored in custom implant fabrication and materials. In the RESORBM initiative, for example, researchers applied machine learning to optimize the design and degradation profile of resorbable molybdenum-alloy implants for pediatric craniofacial reconstruction [10]. By modeling how the implant would gradually dissolve and transfer load to the healing bone, the AI helped in developing a safer resorbable plate that maintains strength during the critical healing period and then disappears, obviating the need for a removal surgery. This kind of AI-driven materials innovation is complemented by work on reducing imaging artifacts: algorithms have been used to evaluate alternative implant materials such as PEEK and ceramic composites, which cause fewer distortions on postoperative CT/MRI, thereby improving postoperative monitoring [10]. While these applications are specialized, they underscore a guiding principle: AI in OMFS is most effective when tailored to the specialty’s unique anatomical and reconstructive challenges, rather than applying one-size-fits-all solutions [1,12].

#### AI Applications in Diagnosis and Surgical Planning

Beyond image segmentation and visualization, artificial intelligence (AI) increasingly contributes to diagnostic reasoning and the design of surgical plans in oral and maxillofacial surgery (OMFS). Deep learning and multimodal fusion techniques have demonstrated high accuracy in differentiating odontogenic cysts, tumors, and inflammatory lesions using panoramic radiographs, cone-beam CT (CBCT), and MRI, often achieving diagnostic performances exceeding 90% [2,13,14]. In temporomandibular joint (TMJ) analysis, automated classification of internal derangements and degenerative changes has been shown to support early, objective diagnosis and facilitate targeted therapeutic planning [13,14,15]. Such systems not only reduce observer variability but can also flag regions of uncertainty, assisting surgeons in complex or ambiguous cases.

In addition to diagnostic assistance, AI is now deeply embedded in virtual surgical planning (VSP) workflows. Machine-learning-based algorithms accelerate osteotomy planning, implant design, and soft-tissue simulation, thereby increasing reproducibility and reducing manual workload [6,9,10,12]. Generative design approaches can automatically propose osteotomy lines and patient-specific implant geometries optimized for occlusal stability and nerve preservation [6,10]. Predictive soft-tissue simulation models, capable of sub-millimeter precision, allow preoperative visualization of postoperative facial appearance and have been clinically validated in orbital and orthognathic reconstruction cases [2,12].

Advanced reinforcement-learning frameworks are emerging that iteratively refine surgical plans by optimizing variables such as symmetry, bone contact area, and mechanical stability. These developments mark a transition toward semi-automated, data-driven planning processes where the surgeon remains the ultimate decision-maker but benefits from algorithmic recommendations derived from large surgical datasets.

AI also enables automated design and additive manufacturing of surgical guides and implants. Systems combining deep-learning segmentation with CAD modules can generate patient-specific implants within minutes, reducing overall planning time by up to 40% [10,12]. In craniofacial reconstruction, AI-assisted biomechanical simulations predict stress distribution across reconstructed segments and fixation plates, enhancing implant durability and reducing intraoperative adjustments [6,10].

Despite these advances, most AI-driven diagnostic and planning systems remain in early clinical validation. Current limitations include small, single-center cohorts and lack of standardized evaluation metrics. Multicenter studies and regulatory approval will be crucial to confirm generalizability. Ultimately, AI should be regarded as an augmentative tool—enhancing precision, reproducibility, and efficiency while preserving the surgeon’s expertise, creativity, and responsibility in the diagnostic and planning process [6,10,16].

### 3.2. Risk Prediction

AI for Clinical Decision Support and Risk Prediction (Table 2): Another critical application area is predictive analytics—using AI to support diagnosis and clinical decision-making in OMFS. Machine learning models can analyze complex combinations of clinical data (imaging findings, patient history, lab results, etc.) to identify patterns and predict outcomes that might not be apparent to an individual clinician. For instance, researchers have trained AI algorithms to classify subtypes of temporomandibular joint disorders (TMD) using MRI scans and patient data, achieving high sensitivity and specificity in distinguishing internal derangement patterns [13,14]. Such tools can assist surgeons in diagnosing TMD more accurately and choosing appropriate treatments, thereby reducing diagnostic dilemmas in a domain where subjective interpretation often leads to variability. In the context of dentoalveolar surgery, a deep neural network model was developed to predict the difficulty of third molar extractions and the risk of inferior alveolar nerve injury based on panoramic radiographs [2]. The AI analyzed features like root geometry, bone density, and nerve proximity, and was able to stratify cases by likely extraction difficulty (easy, moderate, high) while flagging those at elevated risk for nerve damage [2]. This kind of risk stratification model serves as a decision aid: for high-risk cases, the surgeon might opt for additional imaging (such as CBCT), informed consent would be particularly emphasized regarding nerve injury risk, and a more experienced surgeon could be assigned if available. Early results from these models are promising, with accuracy levels in pilot studies often exceeding 85–90%, but they require further validation in diverse patient populations before routine clinical adoption.

AI has also shown value in prognostic modeling for complex surgical cases. In microvascular free flap reconstructions (such as mandibular reconstruction with fibula free flap), outcomes can depend on numerous factors including patient comorbidities, nutritional status, flap ischemia time, and more. Multivariate AI models (e.g., using random forests or neural networks) can integrate these variables to predict the risk of complications like flap failure or poor wound healing [10,17]. One experimental model incorporated not only standard clinical factors but also novel inputs like patients’ oral microbiome profiles and nutritional indices to predict wound healing outcomes, reflecting a multimodal AI approach [9,17]. Such a model could identify high-risk patients preoperatively, prompting interventions like nutritional optimization, closer postoperative monitoring, or alternative reconstructive strategies to improve success rates. While these complex models remain under research, they represent a move toward personalized surgery: using big data and AI to tailor treatment plans and perioperative care to the individual patient’s risk profile.

In addition to outcome prediction, AI is making strides in diagnostic assistance for OMFS imaging. Computer vision algorithms, particularly convolutional neural networks, have been applied to radiographs, CTs, and even intraoral photographs to detect pathology. For example, AI models can screen panoramic X-rays for pathologic lesions (cysts, tumors, impacted teeth) with accuracy comparable to oral radiologists [2]. One scoping review identified numerous AI systems capable of diagnosing conditions like periapical radiolucencies, jaw cysts, and maxillary sinusitis on dental radiographs, often with 90%+ accuracy in controlled tests [2]. Incorporating such tools into practice could mean that a preliminary “read” of a new patient’s panorex is available to the surgeon before they even start their own review, possibly highlighting areas of concern (for instance, an asymptomatic cyst) that warrant attention. Another emerging application is improving imaging quality: algorithms have been developed that post-process intraoral or panoramic X-rays to reduce noise and enhance contrast, which can help in spotting fine details [2]. This is especially useful for low-dose imaging or scans taken in suboptimal conditions. In fact, Sankar et al. describe AI software that filters out radiographic artifacts (for example, metal braces or implants causing scatter) to produce clearer diagnostic images, which can be invaluable in surgical planning [10].

Integration with electronic health records (EHRs) is a further aspect of AI-driven decision support. By mining the rich data in EHRs, AI can uncover trends such as which patients are likely to miss appointments, which treatments lead to more follow-up visits, or which patient profiles tend to have complications. For instance, one analysis in general dentistry used AI to identify patients who had unfinished treatment plans or were overdue for follow-up, enabling targeted reminders and significantly improving completion of care [18]. In an OMFS practice, an AI could similarly flag, say, all orthognathic surgery patients who have not had their planned post-operative orthodontic follow-up, or analyze prescription patterns to alert if a patient might benefit from an alternate pain management strategy based on their history. These kinds of insights can support clinical decisions and prompt quality improvements that might be overlooked amidst a busy practice.

Integration with electronic health records (EHRs) is another important aspect of AI-driven decision support. By mining the rich data contained in EHR systems, AI can uncover trends such as which patients are likely to miss appointments, which treatments are associated with higher complication rates, or which patient profiles tend to require unplanned follow-up care. For instance, one analysis in general dentistry used AI to identify patients who had unfinished treatment plans or were overdue for review, enabling targeted reminders and significantly improving completion of care [18]. In an OMFS setting, similar predictive analytics could flag orthognathic surgery patients who have missed postoperative orthodontic appointments or highlight prescribing patterns that warrant adjustment.

These insights can support clinical decisions and prompt quality improvements that might otherwise be overlooked in a busy practice. When appropriately validated and integrated, EHR-based AI systems extend decision support beyond imaging and diagnostics, fostering proactive management of patient care while reducing administrative workload.

It is important to note that while current capabilities are impressive, AI models have limitations that must be acknowledged. Many AI systems perform best under the conditions similar to their training data; if an AI was trained mostly on adults, it might misclassify pediatric cases, for example. Uncommon presentations or atypical anatomies can confuse models that lack diverse training examples. Moreover, most diagnostic AI provides probabilistic outputs—essentially, a “confidence” in a finding—rather than absolute certainty, and these outputs can sometimes be overconfident. Thus, clinicians should use AI as a complement to, not replacement for, their judgment. In summary, AI-driven decision support in OMFS is expanding our diagnostic and prognostic toolkit, augmenting the surgeon’s cognitive capacity by processing vast information quickly [1,17]. When appropriately validated and integrated, these tools can lead to more evidence-based, personalized care, but they must be implemented with a clear understanding of their scope and limits.

**Table 2:** Risk Prediction and Clinical Decision Support.

Domain of Application	Reported Clinical Benefits	Key Limitations	Validation Status
TMD classification (MRI + clinical data) [13,14]	Higher sensitivity/specificity in diagnosis; improved treatment selection	Limited datasets; mostly experimental	Pilot studies; not yet multicenter
Third molar extraction difficulty & nerve injury risk [2]	Stratifies extraction difficulty; flags high-risk cases	Accuracy may vary across populations; not fully validated	Early validation; small sample cohorts
Intraoperative AR & navigation	Real-time guidance, enhanced precision in extractions and reconstructions [1,3]	Experimental; limited availability and high cost	Pilot studies, early-stage prototypes
Custom implant & biomaterials design	Patient-specific prosthetics, safer resorbable implants, reduced imaging artifacts [10]	Specialized applications; lack of large-scale trials	Preclinical and early clinical validation
Imaging pathology detection (cysts, tumors, lesions [2]	Comparable accuracy to oral radiologists (>90% in some tests)	May misclassify atypical/pediatric cases	Scoping reviews; controlled tests only
Imaging enhancement & artifact reduction [2]	Noise reduction, clearer radiographs, better planning	Limited generalizability; vendor-specific	Prototype tools; early adoption
EHR-based predictive analytics [18]	Identifies missed appointments, complications, incomplete treatments	Dependent on data quality and integration	Reported in general dentistry; limited OMFS-specific validation

### 3.3. Administrative Workflow

AI-Enhanced Administrative Workflow in OMFS: AI’s impact in OMFS extends beyond direct clinical care into the administrative workflow of surgical practice. Managing an OMFS clinic involves coordinating appointments, triaging patient inquiries, handling documentation, and numerous other routine tasks that, while essential, do not always require the surgeon’s expertise. By automating or streamlining these processes, AI can free up time and reduce inefficiencies, ultimately allowing the surgical team to focus more on patient care.

Appointment scheduling and no-show reduction is one immediate area where AI has proven beneficial. Traditional scheduling often relies on fixed templates and manual reminders, and missed appointments (“no-shows”) are a persistent problem that leads to wasted time and longer wait lists. AI-driven scheduling systems employ predictive analytics to tackle this issue. By analyzing historical appointment data, machine learning models can predict the likelihood that a given patient will miss their appointment. Relevant factors might include the patient’s past attendance record, the type of procedure, lead time to the appointment, and even weather or traffic patterns on the day. With a predictive model that flags high no-show risk (studies in primary care have achieved ~86% prediction accuracy), staff can intervene proactively—for example, by double-booking that slot, scheduling a backup patient on call, or sending personalized reminder messages to encourage attendance. In one case, implementing an AI model to guide interventions led to a 50% reduction in missed appointments, significantly improving clinic productivity. Although that data comes from a general healthcare setting, the same principles apply to OMFS clinics where surgical consult slots and OR time are precious. Additionally, AI scheduling software can dynamically optimize the calendar: it may suggest shorter follow-up slots for simple cases and longer consults for complex cases based on learned patterns, or rearrange openings to minimize gaps. Over time, a learning system can adapt to an individual practice’s rhythms, for instance recognizing that Monday afternoons have a high cancelation rate and adjusting accordingly. Real-world evidence of AI’s impact on scheduling is accumulating. These improvements highlight how predictive analytics combined with automation can dramatically improve efficiency and access, turning appointment management from a static process into a dynamic, intelligent system.

Patient triage and virtual intake represent another workflow stage ripe for AI enhancement. OMFS practices often need to triage referrals or patient calls to determine who needs urgent care (e.g., facial trauma, severe infections) versus who can be scheduled electively. AI tools, including chatbots and image-recognition algorithms, can assist with this initial screening. For example, AI-driven chatbots can engage patients on a clinic’s website or messaging app, gathering information about their symptoms through a guided conversation. A patient with a painful swollen jaw might be asked a series of questions by the chatbot (using NLP to interpret responses), and based on the patterns recognized—severe pain, fever, rapid swelling—the system can flag the case as urgent, prompting staff to fit that patient in as an emergency appointment [4,5]. Conversely, a patient inquiring about a long-standing jaw asymmetry with no pain might be triaged as routine, and the AI could even help schedule an appropriate consult time. AI can also leverage computer vision in triage: tele-dentistry platforms now allow patients or referring doctors to upload photos or radiographs of a condition (for instance, an intraoral photo of a lesion or a panorex X-ray of a trauma). Machine learning algorithms analyze these images for critical findings—one prototype system could detect mandibular fractures on uploaded X-rays with high sensitivity, alerting the surgeon if an acute fracture is likely present [19]. Another study reported using AI for remote triage of oral lesions, successfully distinguishing potentially malignant lesions from benign ones to prioritize specialist referrals [15]. By performing a preliminary analysis around the clock, these tools ensure that urgent issues are identified promptly, even if they arise during off-hours. Importantly, any AI-based triage is intended to support, not replace, clinical judgment. In practice, this means the AI might sort incoming inquiries into categories (emergency, priority, routine) with a rationale, but a clinician reviews these categorizations each morning or in real-time for emergencies. Clinics that have implemented AI chatbots for intake often find that a large portion of basic questions and appointment requests can be handled automatically, reserving front-desk staff time for more complex coordination. As a side benefit, the structured data gathered by a chatbot (symptoms, duration, relevant medical history) can be piped into the patient’s chart or a waiting list, so that when the surgeon reviews it, they have a concise summary rather than starting from scratch. Overall, AI-assisted triage can streamline the funnel from patient inquiry to scheduled visit, improving safety by catching emergencies and efficiency by automating routine screening.

Managing clinical documentation is another burden where AI can dramatically improve workflow. Oral and maxillofacial surgeons, like all healthcare providers, spend substantial time writing operative notes, consultation summaries, and correspondence. Speech recognition and NLP have advanced to the point that AI-driven transcription tools can draft clinical notes in real time. Surgeons can dictate findings or procedural details verbally (even during an exam or right after surgery), and AI software transcribes it near-instantaneously into text [4]. Modern medical speech recognition systems, often powered by deep learning, have achieved word error rates low enough that many reports need only minimal editing. In an oral surgery practice, this means a surgeon might save 10–15 min per patient that would have been spent typing or handwriting notes—one practitioner reported that switching to voice transcription freed up nearly an hour in a full clinic day [6,7]. Beyond just transcribing, AI can intelligently format and even summarize documentation. For example, if a surgeon dictates an operative report, an advanced NLP system can parse the narrative and organize it into structured sections (Indication, Approach, Findings, Closure, etc.). It might auto-populate standardized phrases and checklists (e.g., “time out performed, antibiotics given”) based on context, ensuring completeness and compliance with documentation standards [7,16]. Some EHR-integrated AI assistants can also pull up relevant patient history in the midst of documentation—for instance, if the surgeon says “The patient’s medical history is significant for.”, the system might retrieve the past medical history from the chart for confirmation or inclusion. Early studies on NLP in healthcare have shown it can extract key information from unstructured notes with high accuracy [9,17], and even summarize patient records for quick review [1]. By automating documentation, AI not only saves time but can improve quality: notes become more standardized and thorough (fewer omissions or illegible scribbles), which aids in communication with other providers and in medicolegal record-keeping. Of course, surgeons need to verify AI-generated text—oversight is necessary to catch any errors in medical terminology or context—but as the technology improves, the edits required have become increasingly minor. Some OMFS clinics have also started exploring AI to assist with coding and billing documentation, where algorithms suggest CPT codes or generate billing summaries from the content of the note, again subject to human confirmation. This can further streamline the workflow by reducing back-and-forth with billing staff for clarifications.

AI can additionally serve as an internal support tool for clinic staff. Consider the myriad of small questions that arise in a practice: “How do I input this insurance pre-authorization in the system?” or “What’s our protocol for patients on blood thinners?” Instead of flipping through manuals or interrupting colleagues, staff could consult an AI-based chatbot trained on the clinic’s own protocols and knowledge base [5,20]. Such an internal AI assistant can be fed with the clinic’s standard operating procedures, EHR user guides, and policy documents. When a staff member asks a question in natural language, the AI quickly provides the answer or step-by-step guidance. This on-demand help can significantly cut down training time for new employees and reduce errors by ensuring everyone has quick access to correct information.

In summary, AI tools targeting administrative workflows address several pain points of OMFS practice: they keep the schedule full and efficient, ensure urgent patients are promptly attended, liberate surgeons and staff from tedious clerical work, and maintain smooth internal operations. The cascading benefit is that by running a more efficient clinic, patients ultimately experience better service—shorter waits, timely responses, and a team that has more bandwidth to focus on their needs. The significant gains reported in appointment adherence and patient satisfaction when AI automation was introduced in OMFS clinics [6] exemplify this outcome. Crucially, these improvements do not come at the expense of personal interaction; rather, by automating the background tasks, AI creates more opportunities for genuine human engagement where it matters most (Table 3).

The reported clinical benefits are derived from published literature unless otherwise indicated. Statements labeled as “authors’ own experience” refer to multicenter observations from OMFS practices in Ulm and Vienna, conducted as part of AI-driven workflow optimization pilots.

**Figure 1:**
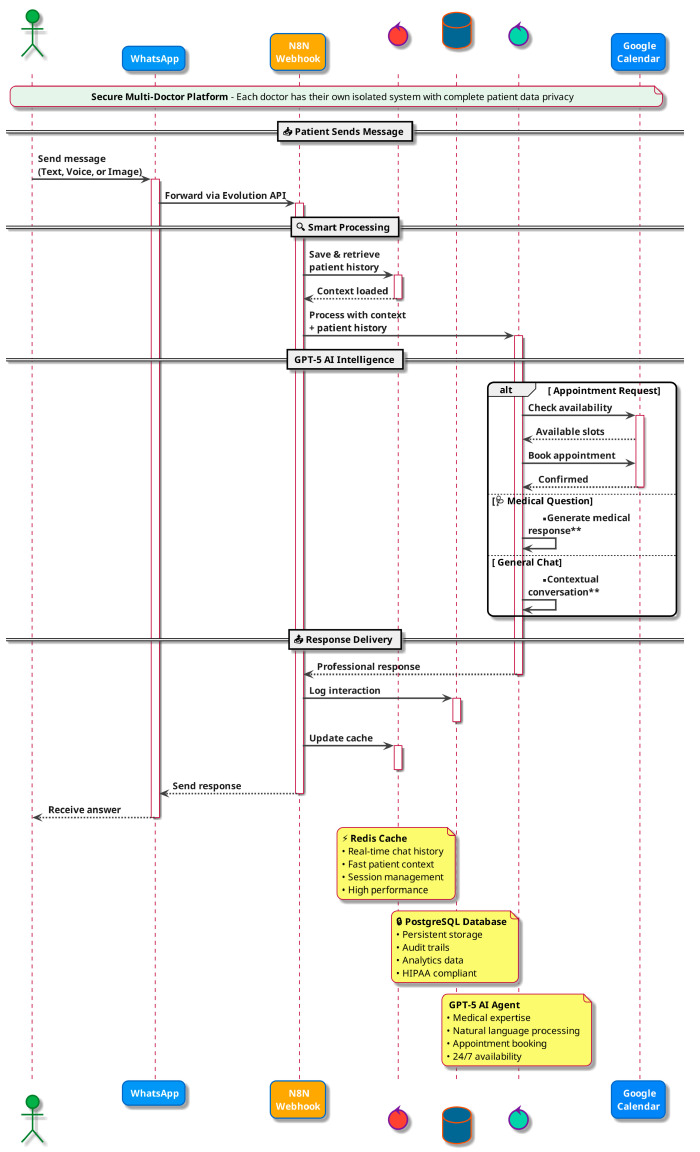
AI-Powered Administrative Workflow System for OMFS Practice Management. ** indicates conditional workflow steps (e.g., human-in-the-loop escalation or manual intervention).

This technical flow figure (Figure 1) demonstrates the complete patient communication pipeline using WhatsApp integration, N8N automation, Redis caching, and GPT-5 AI processing, illustrating how AI can streamline administrative workflows while maintaining professional patient engagement standards in oral and maxillofacial surgery practices.

### 3.4. Patient Communication

AI for Patient Engagement and Communication: Maintaining robust patient engagement is as important to surgical success as the technical execution of procedures. In this context, the term AI co-pilot refers to a conversational support system that assists clinicians during virtual consultations by transcribing dialogue, summarizing key points, and providing context-sensitive prompts or educational material in real time. Patients who are well-informed, comfortable asking questions, and active in their care tend to have better experiences and adherence. AI technologies, especially those leveraging NLP and conversational interfaces, are increasingly being used to enhance patient communication in healthcare. In OMFS—where diagnoses and treatments can be complex and anxiety-provoking—AI offers novel ways to educate and support patients before and after their surgeries.

One of the most visible trends is the use of chatbots and virtual assistants to handle patient queries and routine communication. These AI-driven agents can be deployed on a clinic’s website, social media page, or via messaging apps to provide information 24/7 [4,5]. For example, a prospective patient might visit an OMFS clinic website after hours and interact with a chatbot that answers frequently asked questions: “What services do you offer?”, “Do I need a referral to see the surgeon?”, “How do I prepare for wisdom teeth removal?”, etc. The chatbot uses NLP to understand the question and retrieves a relevant answer from a curated database. Because it responds instantly, patients receive timely information without waiting for office hours, which can reduce frustration and capture potential patients who might otherwise bounce away [4,5]. Moreover, these virtual assistants can personalize the interaction if integrated with scheduling and patient data. For instance, after a patient’s surgery, the system could automatically follow up: “Hello [Name], this is Dr. [X]’s virtual assistant. It’s been 48 h since your surgery—how are you feeling?” Depending on the patient’s response (which the AI can parse for keywords like “pain,” “swelling,” “fine”), it can offer tailored advice or prompt the patient to contact the clinic if certain warning signs are present. This kind of proactive outreach makes patients feel cared for and can catch potential complications early. In one study of a general surgery follow-up chatbot, patients reported higher satisfaction and felt the clinic was more “present” in their recovery, compared to standard care, though objective outcomes were similar [18]. It is important that these chatbots seamlessly hand off to human staff when queries become complex or emotional. For example, if a patient types “I’m really nervous about the anesthesia risks,” the AI should recognize this as a situation that needs a human touch and alert a nurse or surgeon to personally follow up. In our practice, we have found that by handling routine communications (appointments, FAQs, check-ins), AI frees up staff time so that when a patient truly needs personal reassurance or detailed discussion, the team is more available for those high-value interactions.

**Figure 2:**
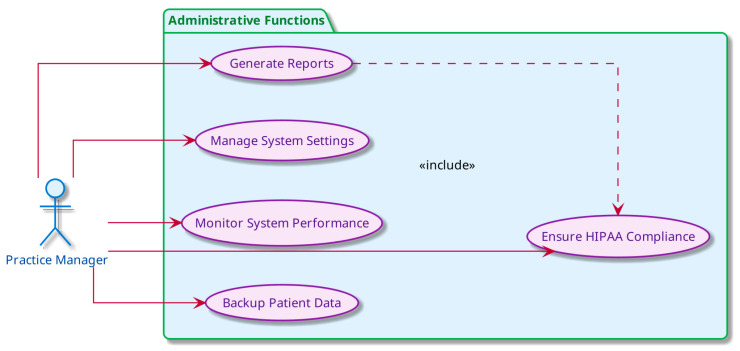

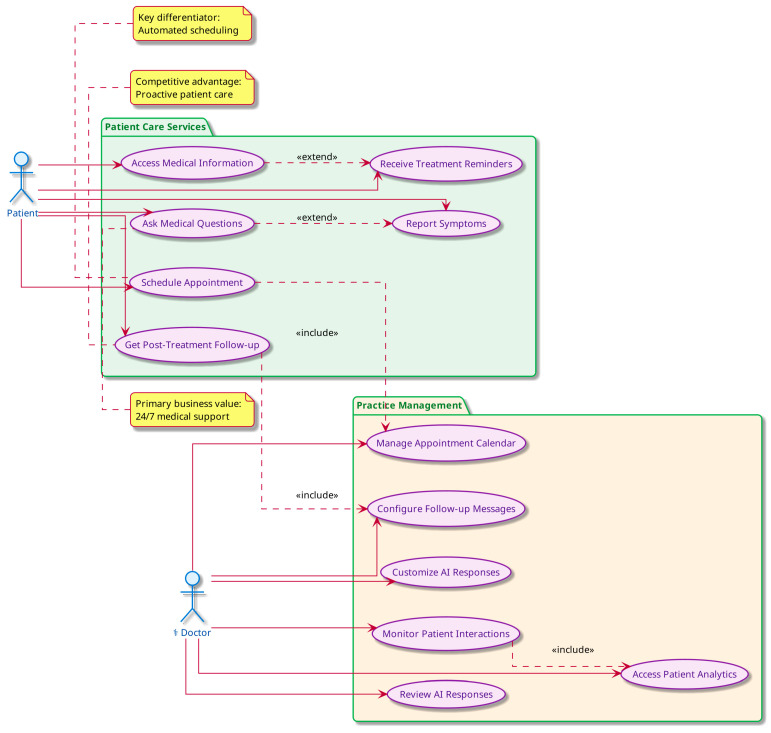
Stakeholder Use Cases for AI Medical Assistant in OMFS Patient Communication.

This business use case (Figure 2) illustrates the multi-stakeholder interactions (Patient, Doctor, Practice Manager) with AI-powered communication systems, demonstrating the comprehensive benefits including 24/7 patient support, automated scheduling, and enhanced practice management that contribute to improved patient satisfaction and reduced administrative burden.

AI is also being leveraged to simplify and personalize patient education. OMFS often involves explaining medical information that can be technical—consider an MRI report describing a condylar tumor, or a detailed post-op care instruction sheet. Many patients struggle with medical jargon and complex explanations. Here, large language models (LLMs) like GPT-5 can act as a real-time translator of medical information. A recent randomized comparative study demonstrated this capability clearly: researchers took actual dental radiology reports and used an AI (ChatGPT 4.0-based) to re-write them in lay-friendly language, then gave patients either the original or AI-simplified report [8,21]. The results were striking—patients who received the AI-simplified reports had much better understanding of their condition and reported feeling significantly more prepared for their consultations [21,22]. The AI-generated text maintained accuracy but used plain language and helpful analogies. Readability scores of the AI reports were far higher (meaning easier to read) than the originals, and patient satisfaction with communication was markedly improved [22,23]. In our context, we can envision using a similar approach for complex surgical explanations: an AI could generate a patient-specific summary of why a particular procedure is recommended and what it entails, which the surgeon can review and hand to the patient as a reference [11]. This does not replace the informed consent discussion, but it reinforces it in writing, in a form the patient is more likely to understand and keep. Additionally, AI can personalize education to the patient’s level of health literacy or even language preference—for instance, instantly translating and simplifying an English post-op instruction sheet into Spanish at an appropriate reading level. Interactive education is another avenue: patients could ask questions to an AI-powered app like “What does it mean to have an ameloblastoma?” and receive a clear, accurate answer immediately, potentially with images or even a short video if the system has multimedia capability. By making information more accessible, AI tools empower patients, which in turn promotes engagement and adherence. Patients who truly grasp their diagnosis and the rationale for treatment are more likely to follow pre-surgery instructions (like fasting or medication adjustments) and post-surgery care plans, and they tend to experience less anxiety.

Telemedicine and virtual consultations have grown, especially spurred by the COVID-19 pandemic, and AI can augment these remote interactions to improve patient engagement. In a virtual OMFS consultation, an AI system might operate in the background as a “digital co-pilot.” For example, speech recognition can transcribe the conversation in real time, allowing the patient to receive a written summary afterwards (which has been shown to improve recall of medical advice). The AI might also monitor the dialog for specific questions or emotional cues; if a patient says, “I just don’t understand what a Le Fort osteotomy is,” the system could quietly prompt the surgeon with a visual aid or a simpler explanation of that term. Experimental platforms have even proposed that generative AI could suggest personalized treatment options during a consult by analyzing the patient’s data against vast medical literature [6,16]. In oral surgery, one could imagine an AI that, upon hearing a patient’s history of obstructive sleep apnea and malformed jaw, suggests to the surgeon, “Consider discussing maxillomandibular advancement surgery, which has a 90% success rate for similar patients,” along with citations—essentially acting as a live decision-support tool. While this level of integration is still conceptual, a commentary in the IAOMS bulletin dubbed such AI assistance during consults a potential “game-changer” for efficiency and comprehensiveness [16]. On the more immediate horizon, AI is already being used in postoperative follow-up as alluded to above. Automated systems send periodic check-ins and can even capture patient-reported outcomes. For example, after orthognathic surgery, patients might receive a daily prompt asking them to rate pain levels, note if they have fever, and take a selfie to assess swelling. An AI can analyze these inputs—flagging if pain is above a threshold or if swelling appears abnormally increasing—and alert the surgical team for a possible early intervention (like evaluating for infection or hematoma). Simultaneously, it provides reassurance and guidance: if everything is on track, the patient might receive a message like, “Your recovery seems to be progressing normally. Remember to stick to soft foods and do your jaw exercises—it makes a big difference!” Such constant, AI-facilitated engagement keeps patients connected to their care and can improve outcomes by ensuring problems are addressed promptly. A study on an AI postoperative monitoring app found that complication detection times were improved and patients were more likely to reach out for help when prompted by the app’s questions, compared to a control group that only had standard discharge instructions [18].

**Figure 3:**
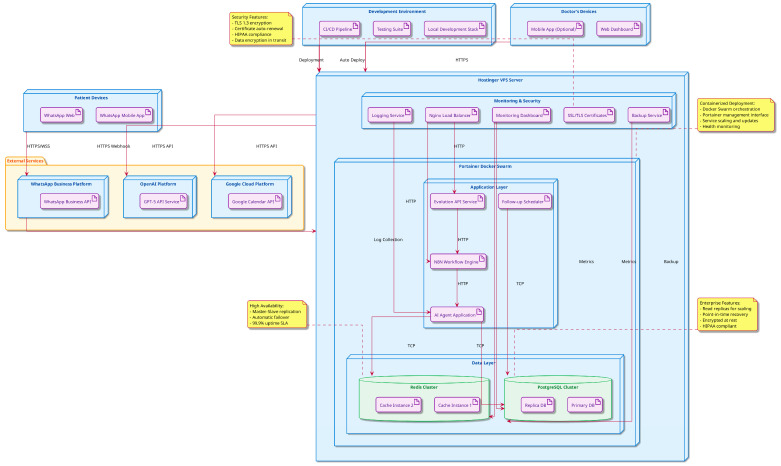
Enterprise Deployment Architecture for AI-Powered OMFS Communication Platform.

A crucial theme in applying AI to patient engagement is preserving the human touch. We must design these tools to complement, not replace, the surgeon–patient relationship built on trust and empathy. As experts have pointed out, automation should serve to enhance the human elements of care, not erode them [20]. In practical terms, this means setting clear boundaries: AI handles the simple, repetitive interactions, while clinicians handle the sensitive, complex ones. Our clinic established protocols (Figure 3) such that if a chatbot detects distress or confusion (through keyword triggers or sentiment analysis), it flags a human team member to personally intervene. Far from depersonalizing care, this strategy has re-personalized it—by offloading mundane tasks (answering the same post-op diet question for the 20th time, for example), the staff now have more time to call patients who need extra support or to sit with a patient an extra 10 min to alleviate their fears. Interestingly, many patients, especially younger ones, appreciate the instant connectivity of AI-driven communication. They will message the virtual assistant at 10 P.M. expecting an answer, something they would not have with a traditional setup, and they are happy with the prompt guidance provided. At the same time, they know that their surgeon is just a phone call away if needed, and that knowledge—combined with the frequent automated check-ins—gives them a strong sense of being cared for.

This human-centered approach to AI deployment requires thoughtful implementation and continuous oversight by clinicians. Surgeons should be transparent with patients that some communications might come through an automated system designed to help them; such transparency actually builds trust, as patients understand that the technology is there to improve their experience, not to cut corners. Indeed, initial feedback from our patients has been positive; they often comment that “the team was really on top of things and always available,” even though some of that availability was via AI assistance. The technology is essentially invisible to them when it is working well—they just feel the clinic is responsive and supportive (Table 4). In conclusion, AI in patient engagement can strike a balance where efficiency and personalization coexist: routine needs are met swiftly by AI, and humans are freed to deliver empathy and expertise where it matters most [20].

## 4. Discussion

The integration of AI into OMFS requires a careful balance: leveraging automation for efficiency and consistency without compromising the empathy and individualized attention that define high-quality care. When thoughtfully implemented, AI can enhance—rather than diminish—the human aspects of clinical interaction, and must remain a tool to assist, not replace, the clinician. In OMFS, where concerns about appearance and function heighten anxiety, trust in the surgeon is essential. Empathy and reassurance cannot be delegated to an algorithm; therefore, AI should be limited to background tasks and first-line communication, while emotional support, complex decisions, and nuanced problem-solving remain under human responsibility.

Despite substantial progress, current AI systems in OMFS still face important limitations. Most models are trained on restricted datasets, which may introduce demographic or anatomical bias and limit generalizability. Pediatric and rare-condition cohorts remain underrepresented in existing datasets, and thus model performance in these populations is uncertain. Furthermore, many deep-learning systems operate as “black boxes,” providing results without transparent reasoning. This lack of interpretability hinders clinical confidence and may impede regulatory approval. Finally, the absence of standardized validation metrics across institutions makes comparative evaluation difficult. Addressing these issues—through dataset diversification, explainable AI frameworks, and multicenter benchmarking—will be essential for trustworthy clinical integration.

By offloading routine work, AI paradoxically creates more time for clinician–patient engagement. If documentation time is reduced by 30% through AI-assisted drafting, that time can be reinvested in follow-ups or detailed consultations. Similarly, when scheduling and reminders are automated, staff are more available for in-person coordination of complex cases. Reports already suggest reduced clerical burden and improved job satisfaction among clinicians using AI tools.

Maintaining a patient-centered model requires transparency and consent. Patients should be informed that the clinic uses advanced systems to improve access and responsiveness—for instance, “a smart scheduling platform and 24/7 virtual assistant.” In practice, most patients respond positively or neutrally to AI involvement as long as it enhances service rather than replacing human contact. Preserving patient choice (e.g., phone calls instead of automated reminders) remains essential.

Equally important is clinical oversight. We treat AI as a junior assistant: capable of significant support but always supervised. Just as a surgeon reviews a resident’s draft, AI outputs are verified before use. This reinforces the ethical and legal principle that ultimate responsibility rests with the licensed professional. Errors can occur—chatbots may misinterpret queries or miss emotional cues. To mitigate this, monitoring tools and staff training are critical.

Adoption of AI in OMFS is influenced not only by technical performance but also by cost-effectiveness and workforce training. Financially, AI systems must demonstrate tangible returns—reductions in missed appointments, improved operating room efficiency, or lower administrative costs—to justify investment. Early pilots in OMFS clinics have already reported substantial gains in patient acquisition and satisfaction at reduced costs, indicating strong economic potential. Equally, workforce training is indispensable: clinicians and staff must learn how to interpret AI outputs, adjust workflows, and maintain ethical standards. Professional curricula should incorporate AI literacy to prepare the next generation of OMFS specialists. Without structured education, even the most advanced tools risk underuse or misuse.

In summary, three principles should guide AI integration in OMFS:

1. Augment, not replace: AI should reduce mechanical burdens and free clinicians to focus on empathy, judgment, and care.
2. Implement responsibly: cost-effectiveness, transparency, and staff training are as important as accuracy metrics.
3. Maintain oversight: ultimate responsibility rests with the surgeon, ensuring technology serves the patient rather than dictates care.

When guided by clinical leadership and ethical diligence, OMFS practices can become more efficient and innovative—yet also more humane. The true promise of AI in healthcare is to restore human connection by reducing mechanical burdens, allowing surgeons to focus on what matters most: healing, communicating, and caring.

The implementation of artificial intelligence (AI) in clinical contexts such as oral and maxillofacial surgery (OMFS) introduces significant ethical and legal challenges. A primary concern is the protection of patient data, as AI systems frequently require access to large datasets—often containing sensitive health information. Compliance with privacy regulations such as HIPAA (USA), GDPR (EU), and Brazil’s LGPD is essential [3,19]. These laws mandate transparency and consent in data usage. For instance, when employing cloud-based AI tools for tasks like image analysis or appointment management, it is imperative that providers ensure encryption, de-identification, and proper access controls. Formal agreements, such as business associate agreements (BAAs), must clearly define data handling protocols and prohibit unauthorized secondary use.

Ethically, transparency with patients is fundamental. Even when data is anonymized, informing patients that their data may contribute to algorithmic improvement maintains trust. Many developers are adopting privacy-preserving strategies, such as local data processing or federated learning. Nonetheless, the responsibility for due diligence rests with clinicians, who must ensure that any AI tool complies with security standards before its integration into care pathways.

Another ethical priority is algorithmic transparency. Many AI models—especially those based on deep learning—function as “black boxes” without interpretable rationales for their outputs. In healthcare, such opacity is problematic. Clinicians must understand the reasoning behind significant AI-driven recommendations [18,19]. For example, AI systems analyzing radiographic images should indicate specific regions of concern, enabling surgeon verification. Similarly, predictive models for appointment no-shows should disclose contributing variables. Explainable AI not only supports clinical confidence but also facilitates communication with patients. Emerging regulatory frameworks, such as the proposed EU AI Act, emphasize explainability as a requirement for high-risk medical applications [19]. The U.S. FDA has also issued guidance on the importance of interpretability in AI medical devices. Surgeons must advocate for tools that allow them to remain informed, autonomous decision-makers.

Accountability in AI-assisted care remains with the clinician. Legally and ethically, AI is an adjunct—not a substitute—for clinical judgment [18]. If an AI tool suggests early discharge, for instance, the final decision still depends on the surgeon’s assessment of clinical stability. Consequently, OMFS training must include instruction on interpreting and critiquing AI outputs. Documentation of AI usage (e.g., “AI risk model indicated high apnea risk; ICU admission elected”) reinforces that clinical oversight guided the decision. Professional associations are beginning to draft guidelines around AI utilization; adherence to such standards will be increasingly important.

Bias and fairness are critical concerns in AI development and deployment. Models trained on homogeneous datasets may underperform across diverse populations. For example, an AI system trained predominantly on adult males may yield suboptimal predictions for females or minority groups. In OMFS, this can manifest as inaccuracies in diagnostic tools or scheduling algorithms. Practices should prioritize tools validated on demographically representative datasets and regularly audit for disproportionate outcomes. Addressing sources of bias—such as geographic or socioeconomic proxies—is essential to ensure equitable care. Organizations such as the ADA have called for transparent performance metrics and fairness safeguards in dental AI systems [19].

Regulatory oversight is evolving. Tools used in OMFS, such as diagnostic imaging software, may require formal clearance. Preference should be given to systems with regulatory approval or published clinical validation. The use of unvalidated tools may represent a deviation from the standard of care and expose practitioners to legal risk. Guidance from entities such as the ADA and AAOMS is expected to become more detailed, including recommendations on practitioner training, FDA clearance, and intended use. Institutions may adopt internal AI governance protocols, reviewing new tools prior to implementation and continuously monitoring their performance. Staying informed about legal precedents will be vital, especially as case law around AI in healthcare develops.

In summary, ethical and legal stewardship of AI in OMFS must be grounded in established principles: respect for autonomy and privacy, beneficence, non-maleficence, and justice. Compliance with data protection laws, commitment to transparency and explainability, rigorous oversight, and attention to equity are essential. As surgeons, we now assume responsibility not only for operative excellence but also for the conscientious integration of emerging technologies into patient care.

Despite the vast potential of AI in OMFS, it is essential to recognize its current limitations and maintain realistic expectations. Many AI applications discussed—ranging from diagnostic models to chatbots—remain in early stages of adoption within OMFS. Real-world performance often diverges from results reported in pilot studies. For example, an AI model with 95% accuracy for detecting jaw fractures in a curated dataset may underperform in a trauma center with variable image quality and atypical cases. Generalizability remains a concern: models trained on specific populations or devices may falter elsewhere. Local validation is thus critical. In our practice, we compare AI outputs against clinician assessments during initial deployment. Discrepancies must be addressed, either by retraining with diverse data or adjusting clinical use.

Another limitation involves the opaque nature of many AI systems. While some models offer explainability, others deliver only a score or binary output. Surgeons may hesitate to trust such tools. In these cases, we treat AI suggestions as supplementary evidence—integrating them with clinical signs, test results, and judgment. Moreover, current AI lacks contextual understanding and common sense. A human surgeon might interpret a patient’s pale appearance or anxious behavior meaningfully; an AI typically cannot. In one case, a chatbot failed to recognize that a patient repeatedly asking minor questions was actually seeking reassurance. The system answered each query but did not escalate the case. This underlines the need for human oversight. Although sentiment analysis and similar tools may evolve, present systems often miss such subtleties.

Technically, training robust AI requires large, high-quality datasets. OMFS lacks the data volume of broader fields like radiology. Some AI tools are thus trained on limited datasets, reducing reliability. Initiatives such as multi-center collaborations are beginning to address this. Approaches like federated learning, which allows training across centers without data sharing, may further enhance generalizability in the future. Until then, edge cases—rare pathologies or anatomical extremes—require caution.

Integration into clinical workflows presents another challenge. Adoption is gradual. Staff require training, and changes in routine may face resistance. We implemented AI features incrementally and designated “AI champions” to assist peers. Initial feedback suggested that checking AI-generated notes took as long as manual writing. With repeated use, however, efficiency improved. This learning curve necessitates structured training and progressive implementation.

Patients also exhibit varying levels of comfort with AI. While younger patients readily engage with chatbots, some older individuals prefer human interaction. Trust may be a barrier: “I want a person, not a machine.” We mitigate this by emphasizing professional oversight and tangible benefits like reduced waiting times. Maintaining alternative communication pathways (e.g., phone calls, printed material) remains necessary.

Nevertheless, AI’s trajectory in OMFS points toward deeper integration. In surgical planning, future systems may optimize osteotomies in real-time, balancing nerve risk and occlusal stability. Generative models could simulate entire surgeries for resident training, offering adaptive challenges and feedback. Haptic simulators powered by AI may revolutionize procedural education. In patient care, multimodal AI could interact via avatars, analyze facial expressions, and guide rehabilitation (e.g., counting mouth-opening exercises via app-based coaching).

Healthcare infrastructure is evolving into a “learning health system,” where outcomes feed continuous AI refinement. OMFS departments embracing this model may uncover best practices from aggregated data—e.g., patterns improving flap success rates. AI may eventually predict intraoperative complications or optimize OR schedules based on historical data, improving efficiency.

Research horizons are broad. AI is being explored to predict adolescent facial growth, improve clarity of consent forms using NLP, and automate 3D printing of surgical guides post-approval. Ethical considerations include embedding informed consent into AI interactions—still speculative, but indicative of ongoing inquiry into AI’s role even in humanistic aspects of care.

A consensus is forming: for AI to benefit OMFS, collaboration between clinicians and developers is essential [5,20]. Surgeons must participate in tool design to ensure clinical relevance and workflow integration. Conversely, basic data literacy may become necessary for future surgeons. Encouragingly, professional societies are beginning to address AI in events and guidelines. As authors, we advocate active engagement with AI—treating it not as a threat, but as a tool akin to virtual planning or endoscopy, to be mastered for patient benefit.

In conclusion, while limitations remain, they are not insurmountable. Through iterative development, robust validation, and thoughtful integration, AI’s contribution to OMFS can become substantial. History shows that transformative technologies—antibiotics, microsurgery, digital imaging—faced skepticism before becoming standards. AI follows a similar trajectory, but its scope encompasses the entire care ecosystem. Successful adoption demands parallel advancements in infrastructure, education, and ethics. With structured evaluation and open reporting of both successes and failures, the OMFS community can ensure AI implementation remains evidence-based and aligned with our shared goal: delivering excellent, compassionate care.

### Clinical Implication

From a practical standpoint, the current evidence supports selective but meaningful clinical use of AI in OMFS. Systems for automated image segmentation, surgical planning assistance, and administrative scheduling have demonstrated tangible benefits in efficiency and accuracy. However, all AI-assisted processes must operate under direct clinical oversight. Surgeons remain responsible for validating AI outputs, particularly in diagnostic and intraoperative contexts. Institutions adopting AI should ensure structured staff training, transparency in patient communication, and continuous monitoring of performance metrics. In its present stage, AI should be viewed as a cognitive and operational aid—enhancing, but never replacing, professional judgment and patient-centered care.

## 5. Conclusions

Artificial intelligence is emerging as a transformative force in oral and maxillofacial surgery, with growing evidence that it can enhance diagnostic accuracy, surgical planning, workflow efficiency, and patient engagement. Its role, however, is to augment—not replace—the surgeon, reducing mechanical burdens while preserving empathy and clinical judgment. Responsible adoption demands attention to cost-effectiveness, structured training, and rigorous validation across diverse settings, always under clinician oversight. When integrated thoughtfully, AI has the potential to make OMFS more precise, efficient, and humane—restoring human connection by allowing surgeons to focus on what matters most: healing, communicating, and caring. Additional figures illustrating the system architecture and workflows are provided in Appendix A.

## Figures and Tables

**Table 1:** Imaging and Planning Applications of AI in OMFS.

Domain of Application	Reported Clinical Benefits	Key Limitations	Validation Status
Automated imaging segmentation (CT, CBCT, MRI)	Faster landmark identification, reduced manual workload, improved consistency [1,9]	Dependent on dataset quality; less robust in atypical cases	Early clinical validation; small cohorts
Virtual surgical planning (VSP) & generative design	Sub-millimeter prediction of soft tissue changes, improved implant fit, reduced intraoperative adjustments [2,10,12]	Limited multicenter and pediatric data	Reported in >100 clinical cases (e.g., orbital floor reconstructions)
Intraoperative AR & navigation	Real-time guidance, enhanced precision in extractions and reconstructions [1,3]	Experimental; limited availability and high cost	Pilot studies, early-stage prototypes
Custom implant & biomaterials design	Patient-specific prosthetics, safer resorbable implants, reduced imaging artifacts [10]	Specialized applications; lack of large-scale trials	Preclinical and early clinical validation

**Table 3:** Administrative Workflow Applications of AI in OMFS.

Domain of Application	Reported Clinical Benefits	Key Limitations	Validation Status
Patient triage & virtual intake [4,5,15,19]	Faster emergency detection; automated sorting of routine cases	Requires clinical oversight; risk of misclassification	Early prototypes; small-scale studies
Automated documentation & transcription [1,4,6,7,9,16,17]	Saves 10–15 min per patient; standardized notes; improved compliance	Needs human review; terminology/context errors	Early adoption; healthcare pilot studies
Internal staff support chatbots [5,20]	Quick access to protocols; reduced training time; fewer errors	Limited validation; dependent on knowledge base quality	Prototype implementations
Operational analytics (e.g., wait times, cancelations) [5,20]	Real-time monitoring; proactive problem-solving	Still experimental; integration challenges	Research/early pilot stage

**Table 4:** Patient Engagement & Communication.

Domain of Application	Reported Clinical Benefits	Key Limitations	Validation Status
Chatbots & virtual assistants [4,5,18]	24/7 responses; faster answers; higher patient satisfaction	Must hand off complex/emotional cases; risk of misunderstanding	Early clinical deployments; mixed-methods evaluations
AI-simplified reports & education [8,21,22,23]	Better understanding; higher preparedness; improved satisfaction	Needs clinician review for accuracy; variable literacy levels	Randomized/controlled usability studies
Telemedicine “AI co-pilot” & decision support [6,16]	Real-time transcription/prompts; more comprehensive consults	Largely conceptual/prototype; integration burden	Commentaries, pilot platforms
Postoperative monitoring & PROs [18]	Earlier complication flags; improved outreach	False positives, alert fatigue	Prospective app-based studies
Human-centered safeguards [20]	Preserves trust; clear boundaries for automation	Requires governance and training	Expert guidance, implementation reports

**Figure A1:**
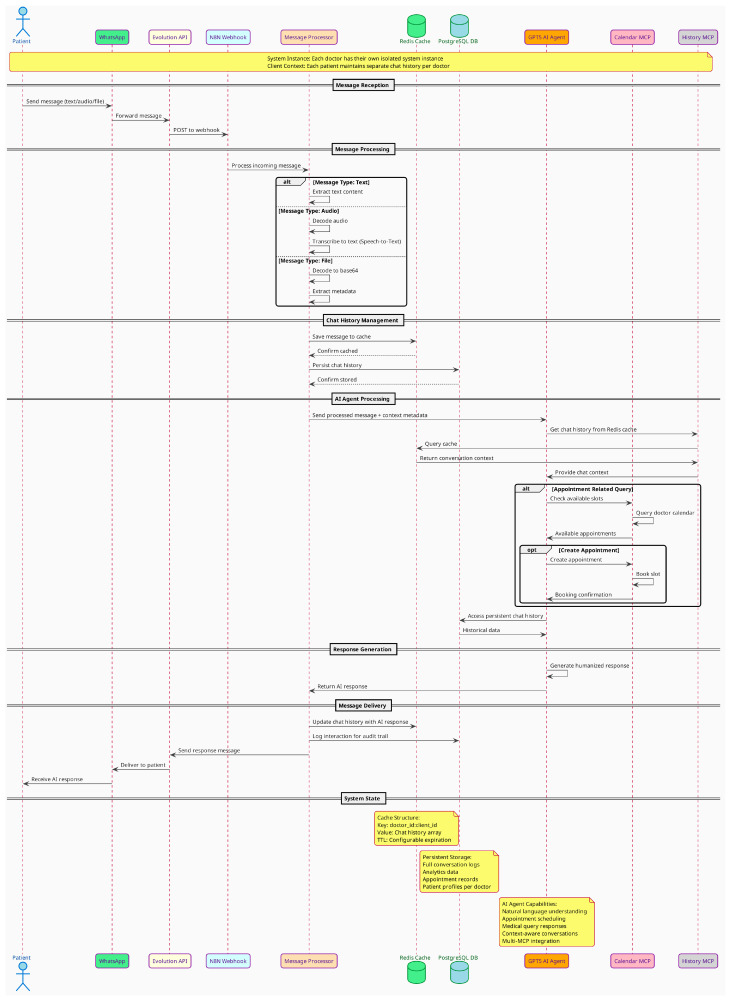
Complete AI Chatbot System Architecture—Detailed technical flow for oral and maxillofacial practice management.

**Figure A2:**
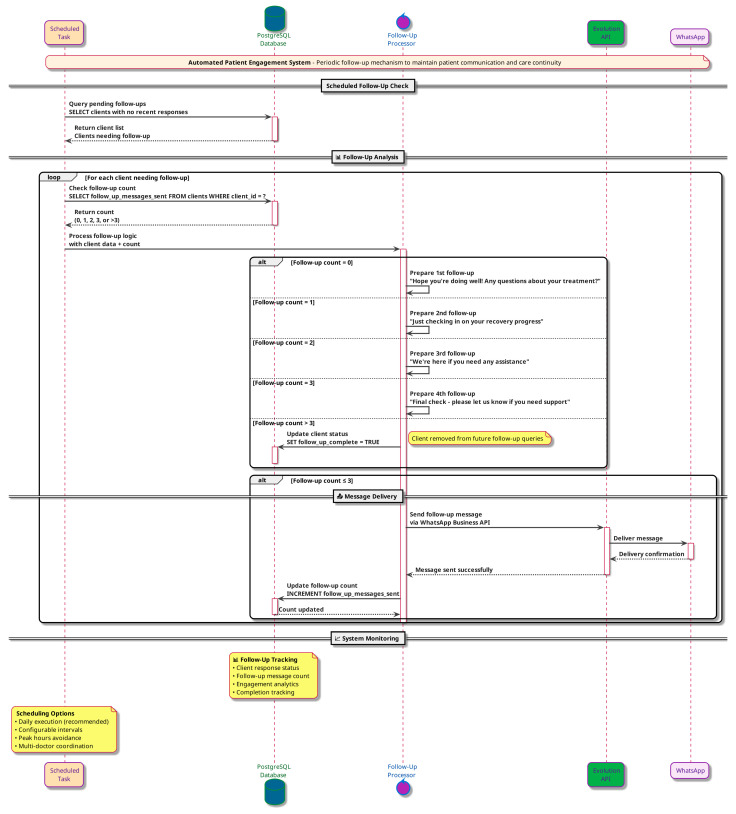
Automated Follow-Up Mechanism—Patient Engagement and Care Continuity.

**Figure A3:**
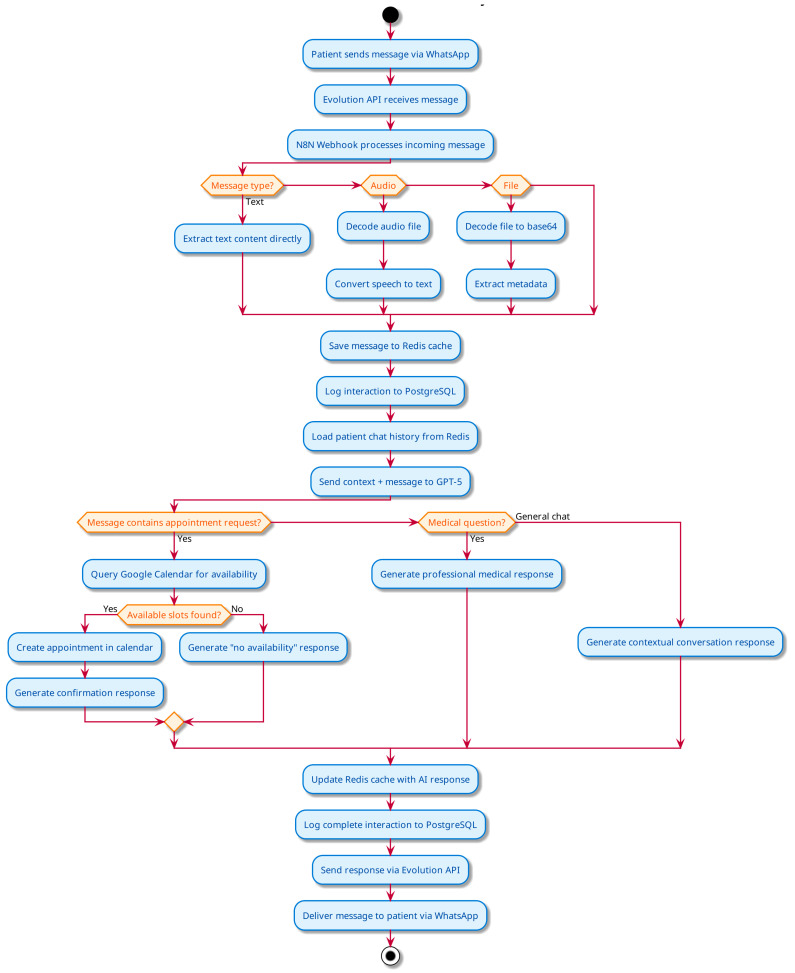
AI Chatbot Main Process Activity Figure—Business Logic and Decision Flow.

**Figure A4:**
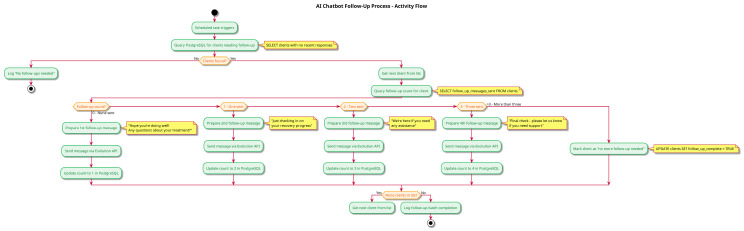
Follow-Up Activity Figure—Progressive Patient Engagement Workflow.

**Figure A5:**
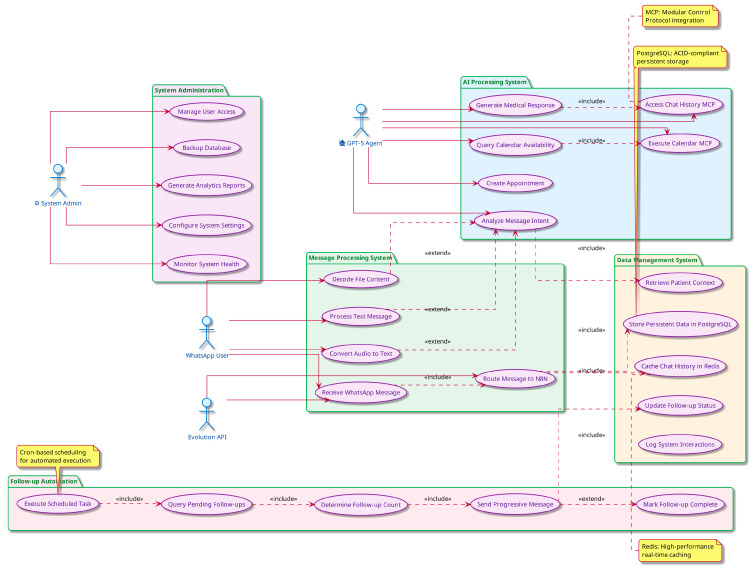
System Use Cases—AI Chatbot Technical Implementation.

## Data Availability

The original contributions presented in this study are included in the article. Further inquiries can be directed to the corresponding author(s).
